# Once-Weekly Semaglutide in Patients with Cardiovascular-Kidney-Metabolic Syndrome: A Real-World Study

**DOI:** 10.3390/ph19040583

**Published:** 2026-04-07

**Authors:** Alicia Trenas-Calero, Nuria Prieto-Laín, Ana I. Gómez-Hernández, Miguel A. Pérez-Velasco, María-Rosa Bernal-López, María-Dolores López-Carmona, María-Dolores García de Lucas, Ricardo Gómez-Huelgas, Luis M. Pérez-Belmonte

**Affiliations:** 1 Servicio de Medicina Interna, Hospital Regional Universitario de Málaga, Instituto de Investigación Biomédica de Málaga (IBIMA), Universidad de Málaga, 29010 Málaga, Spain; aliciatrenascalero@gmail.com (A.T.-C.); prietolain@hotmail.com (N.P.-L.); anaisabelgomezhernandez96@gmail.com (A.I.G.-H.); miguelpv89@gmail.com (M.A.P.-V.); robelopajiju@yahoo.es (M.-R.B.-L.); mdlcorreo@hotmail.com (M.-D.L.-C.); gdelucaslola@gmail.com (M.-D.G.d.L.); ricardogomezhuelgas@hotmail.com (R.G.-H.); 2 Centro de Investigación Biomédica en Red Fisiopatología de la Obesidad y Nutrición (CIBERobn), Instituto de Salud Carlos III, 28029 Madrid, Spain; 3 Departamento de Medicina y Dermatología, Facultad de Medicina, Universidad de Málaga (UMA), Instituto de Investigación Biomédica de Málaga (IBIMA), 29590 Málaga, Spain; 4 Centro de Investigación Biomédica en Red Enfermedades Cardiovasculares (CIBERCV), Instituto de Salud Carlos III, 28029 Madrid, Spain

**Keywords:** semaglutide, cardio-kidney-metabolic syndrome, heart failure, chronic kidney disease, obesity, type 2 diabetes

## Abstract

**Introduction and Objectives:** There is limited evidence on the role of glucagon-like peptide-1 receptor agonists in the interplay between cardiovascular disease, chronic kidney disease, and metabolic dysfunction. This work analyzed the efficacy and safety of once-weekly semaglutide in patients with cardiovascular-kidney-metabolic syndrome. **Patients and Methods:** This observational, real-world study included patients with heart failure, chronic kidney disease, obesity, and type 2 diabetes mellitus treated with once-weekly semaglutide (Sema-CKM Group) and patients not treated with glucagon-like peptide-1 receptor agonists (Control-CKM Group). A 1:1 propensity score matching analysis was performed. The two primary outcomes were heart failure events and major kidney disease events at 24 months. **Results:** After matching, 302 patients were included in each group. A heart failure event occurred in 63 patients (20.9%) in the Sema-CKM Group and 121 (40.1%) in the Control-CKM Group (OR: 0.80; 95%CI: 0.62–0.98; *p* < 0.01). The number of major kidney disease events was lower in the Sema-CKM Group than the Control-CKM Group (36 vs. 65; OR: 0.85; 95%CI: 0.72–0.98; *p* = 0.014). Patients in the Sema-CKM Group were more likely to have an improvement in heart failure health status from baseline to 24 months (OR: 2.80; 95%CI: 1.30–4.30; *p* < 0.01). Semaglutide also improved glycemic control (glycated hemoglobin −0.7%) and reduced body weight (−9.3 kg). **Conclusions:** Once-weekly semaglutide was associated with reductions in heart failure events and major kidney disease events in patients with heart failure, chronic kidney disease, obesity, and type 2 diabetes mellitus. Further research on glucagon-like peptide-1 receptor agonists in cardiovascular-kidney-metabolic syndrome is needed.

## 1. Introduction

There are complex interactions among the cardiovascular, kidney, and metabolic systems. Disorders in these systems—primarily heart disease, kidney disease, diabetes, and obesity—are referred to as cardiovascular-kidney-metabolic (CKM) syndrome. They are driven by excess and/or dysfunctional adipose tissue, insulin resistance, and chronic inflammation [1]. Dysfunction in one of these systems spurs dysfunction in another due to their reciprocal associations. CKM syndrome entails a significant global morbidity and mortality burden [2].

In recent years, sodium–glucose cotransporter 2 inhibitors (SGLT-2i) have been associated with significant cardiovascular, renal, and metabolic benefits, consistently reducing HF hospitalizations, slowing renal disease progression, and providing atherosclerotic cardiovascular protection across the spectrum of CKM syndrome [3,4]. On the other hand, the benefits of glucagon-like peptide-1 receptor agonists (GLP-1ra) on cardiovascular-renal outcomes have also been shown in patients with type 2 diabetes mellitus (T2DM) [3,5]. Semaglutide has been recently associated with benefits on patients with HF with preserved ejection fraction and obesity with and without T2DM [6,7]. Furthermore, other newly developed drugs such as tirzepatide have been associated with remarkable metabolic benefits in patients with T2DM, obesity, or HF [8,9].

Although semaglutide has been associated with relevant benefits in several patients with different cardio-reno-metabolic profiles [3,4,5,6,7], no specific data exists on its role in patients with CKM syndrome. Due to this lack of evidence and the widespread use of semaglutide in our clinical setting, this work aimed to analyze the efficacy of once-weekly semaglutide on cardiovascular/renal outcomes and safety in patients with HF, CKD, obesity, and T2DM. It was hypothesized that the use of once-weekly semaglutide would be associated with reductions in adverse cardiovascular/renal outcomes and would be safe in patients with HF, CKD, obesity, and T2DM.

## 2. Results

A total of 754 patients with CKM syndrome were included in this study. Of them, 388 patients who received once-weekly semaglutide (Sema-CKM Group) and 366 patients who did not receive any GLP-1ras (Control-CKM Group) completed 24 months of follow-up. After PSM, 302 patients were included in each group. At 24 months, a total of 242 patients (80.1%) had reached the dose of 1.00 mg of semaglutide. During observation period, all patients remained under follow-up. The only discontinuations corresponded to deaths.

Table 1 shows the baseline sociodemographic, clinical, and therapeutic characteristics of patients. Before PSM, the proportion of patients with HbA1c < 7% was lower in the Sema-CKD Group than in the Control-CKD Group (3.6% vs. 19.9%, *p* = 0.015) and the HbA1c level was higher (8.1 ± 1.5 vs. 7.2 ± 1.1%, *p* = 0.039). No other differences were found between groups. After PSM, both groups were well-balanced and no differences were found.

**Figure 1:**
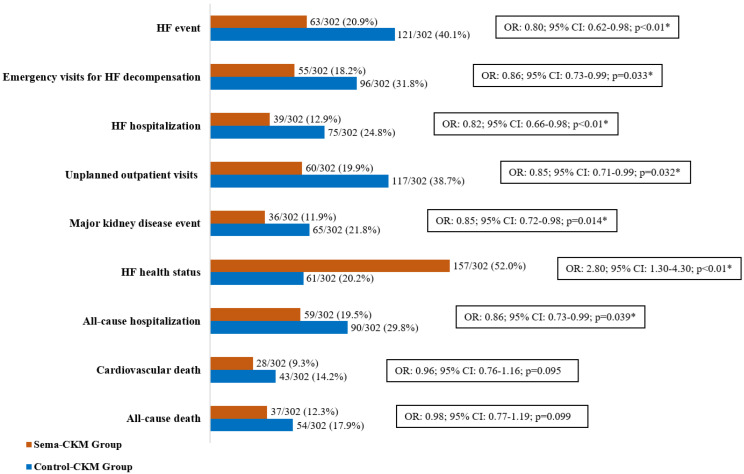
Primary and secondary outcomes. Data are shown as percentages. In order to evaluate the association between treatment and study outcomes, mixed-effect logistic regressions were used. The regression analysis values were expressed as odds ratio and 95% confidence interval. Values were considered to be statistically significant when *p* < 0.05. * Statistically significant (*p* < 0.05). 95% CI: 95% confidence interval; CKM: cardiovascular-kidney-metabolic; HF: heart failure; OR: odds ratio.

**Figure 2:**
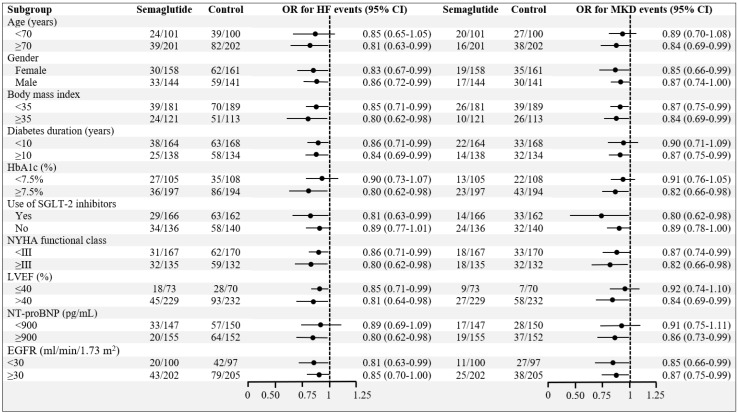
Subgroup analysis of primary outcomes. Data are shown as absolute values (number of patients with events/number of patients at risk). In order to evaluate the association between treatment and study outcomes, mixed-effect logistic regressions were used. The regression analysis values were expressed as odds ratio and 95% confidence interval. Values were considered to be statistically significant if *p* < 0.05. 95% CI: 95% confidence interval; EGFR: estimated glomerular filtration rate; HbA1c: glycated hemoglobin; HF: heart failure; LVEF: Left ventricular ejection fraction; MDK: major kidney disease; NT-proBNP: N-terminal pro-brain natriuretic peptide; NYHA: New York Heart Association; SGLT-2: sodium–glucose cotransporter 2.

A HF event occurred in 63 patients (20.9%) in the Sema-CKM Group and 121 (40.1%) in the Control-CKM Group (OR: 0.80; 95% CI: 0.62–0.98; *p* < 0.01) at 24 months. There were also reductions in their individual components: emergency department visits for HF decompensation (OR: 0.86; 95% CI: 0.73–0.99; *p* = 0.033), HF hospitalizations (OR: 0.82; 95% CI: 0.66–0.98; *p* < 0.01), and unplanned outpatient visits (OR: 0.85; 95% CI: 0.71–0.99; *p* = 0.032). There were fewer major kidney disease events in the Sema-CKM Group than in the Control-CKM Group (36 vs. 65; OR: 0.85; 95% CI: 0.72–0.98; *p* = 0.014). Patients in the Sema-CKM Group were more likely to have an improvement in HF health status from baseline to 24 months (OR: 2.80; 95% CI: 1.30–4.30; *p* < 0.01), with the KCCQ total symptom score increasing to 20.5 (4.0) points in the Sema-CKM Group versus 7.0 (1.5) points in the Control-CKM Group (*p* < 0.01). Furthermore, there was a significant decline in all-cause hospitalizations (OR: 0.86; 95% CI: 0.73–0.99; *p* = 0.039). No significant reductions were observed in cardiovascular or all-cause deaths. Primary and secondary outcome results are shown in Figure 1. The effects of semaglutide on HF events and major kidney disease events appeared to be consistent across the prespecified subgroups (Figure 2).

Patients in the Sema-CKM Group had a bigger reduction in HbA1c (an intergroup difference of −0.7%, *p* = 0.012) and reduced body weight (an intergroup difference of −9.3 kg, *p* < 0.01) than patients in the Control-CKM Group. There were negative correlations between the KCCQ total symptom score and the body weight (r = −0.689, *p* < 0.01) and HbA1c (r = −0.558, *p* = 0.018).

In regard to safety, serious adverse events were observed in 78 patients who received semaglutide (25.8%). All events were gastrointestinal (43 nausea, 21 vomiting, and 14 diarrhea) and occurred during the first few weeks of treatment or after dose increases. Thirty-three patients (10.9%) had more severe gastrointestinal symptoms and discontinued semaglutide.

## 3. Discussion

This study found that the use of semaglutide 1.00 mg once weekly was associated with reductions in HF events and their individual components (emergency department visits for HF decompensation, HF hospitalizations, and unplanned outpatient visits) as well as major kidney disease events in patients with CKM syndrome. Patients treated with semaglutide were also more likely to have an improvement in HF health status and there were fewer all-cause hospitalizations. Patients treated with semaglutide had a bigger reduction in HbA1c and reduced body weight than patients without GLP1-ras. The tolerability and safety profiles were good, with only a few gastrointestinal adverse events.

In recent years, SGLT-2is have shown enormous cardiovascular, renal, and HF benefits in patients with T2DM at high risk for cardiovascular disease or with established cardiovascular disease [3,4]. These benefits have also been shown in patients with HF with reduced and preserved LVEF [10,11,12,13] as well as in patients with CKD regardless of the presence of T2DM [14,15,16,17]. They are recommended as first-line therapy to reduce cardiovascular-renal events [18,19]. Due to the fact that these benefits are mediated by mechanisms that go beyond glucose control, including natriuresis, blood pressure reduction, weight loss, and anti-inflammatory actions, SGLT-2is have been established as pivotal therapy for the management of CKM syndrome [20,21].

On the other hand, GLP-1ras have also demonstrated significant cardiovascular and renal benefits in patients with T2DM, including a reduction in HF hospitalizations, although the effect was modest [5]. Semaglutide has demonstrated significant benefits across the cardiac, renal, and metabolic domains in patients with T2DM, CKD, and/or obesity. In patients with T2DM and CKD, subcutaneous semaglutide 1.00 mg once weekly significantly reduced the risk of major kidney disease events by 24%, slowed eGFR decline, and reduced the risk of major adverse cardiovascular events and all-cause mortality [22]. In patients with obesity and established cardiovascular disease but without diabetes, semaglutide 2.4 mg once weekly reduced major adverse cardiovascular events by 20% and slowed the risk of kidney disease progression [23,24]. Semaglutide has also been associated with improvements in HF outcomes in patients with T2DM and CKD, reducing the risk of HF events and cardiovascular death [25]. The metabolic benefits of semaglutide include improved glycemic control, weight loss, and reductions in blood pressure and albuminuria, all of which contribute to slowing CKD progression and reducing cardiovascular risk. These effects are consistent across subgroups, including those with and without baseline HF or reduced eGFR [22]. Recently, the GLP-1ra efpeglenatide has been associated with a significant reduction in HF hospitalizations in patients with T2DM and either a history of cardiovascular disease or current kidney disease [26]. In regard to HF, to date, only the STEP-HFpEF DM and STEP-HFpEF Trials have specifically evaluated the impact of semaglutide 2.4 mg once weekly on HF outcomes in patients with HF with preserved LVEF. Semaglutide showed reductions in symptoms and physical limitations and improvements in exercise function in patients with HFpEF and obesity with and without T2DM [6,7]. These benefits of subcutaneous and oral semaglutide on HF outcomes have also been shown in several real-world clinical studies in patients with HF with reduced and preserved LVEF, T2DM, and obesity [27,28,29,30]. Furthermore, tirzepatide, a newly developed dual glucose-dependent insulinotropic peptide (GIP) and GLP-1ra, has been associated with remarkable metabolic benefits in terms of sustainable weight loss and glycemic control in patients with T2DM or obesity [8]. Tirzepatide has also been shown to reduce mortality from cardiovascular causes or worsening HF and improve health status in patients with HF with preserved ejection fraction and obesity [9]. In line with all this evidence, this study provides further data on the role of the GLP-1ra semaglutide in CKM syndrome, showing reductions in HF and major kidney disease events in patients with concomitant HF, CKD, T2DM, and obesity.

The cardiovascular-kidney-metabolic benefits associated with GLP-1ras may have multiple underlying pleiotropic mechanisms [31,32,33,34]. GLP-1ras have been associated with metabolic actions through enhancing glucose-dependent insulin secretion, suppressing glucagon release, delaying gastric emptying, and reducing appetite, leading to improved glycemic control and significant weight loss. These effects reduce insulin resistance and mitigate metabolic risk factors [32]. GLP-1ras can also lower blood pressure, improve lipid profiles, and exert anti-inflammatory and antioxidant effects. The cardiovascular mechanisms associated include both indirect effects (via metabolic improvements) and direct actions on the heart and vasculature. GLP-1ras can act on endothelial cells to enhance nitric oxide production and promote vasodilation, such as improved endothelial function and reduced atherogenesis. They modulate atherosclerotic plaque stability by reducing matrix metalloproteinase activity, potentially preventing fibrous cap degradation. GLP-1ras also act on monocytes, macrophages, and foam cells to reduce inflammatory cytokine secretion and inhibit smooth muscle cell proliferation. Other direct vascular effects include the reduction of systolic blood pressure, atherogenic lipoproteins, and inflammatory markers [33]. In regard to renal protection, GLP-1ras reduce albuminuria and slow eGFR decline in a manner that is partly independent from glycemic control and attributed to reduced glomerular hyperfiltration, anti-inflammatory effects, and decreased oxidative stress [32]. GLP-1ras suppress pro-inflammatory pathways including the receptor for advanced glycation end products and Toll-like receptors, potentially through CNS-mediated parasympathetic and opioid efferent activity [33]. By attenuating postprandial glucose and lipid excursions, GLP-1ras can decrease endothelial dysfunction, inflammation, and thrombogenicity, which are key drivers of cardiovascular and kidney injury in diabetes and metabolic syndrome [34]. Additionally, GLP-1ras may contribute to promoting natriuresis, reducing hypertension, and preventing thrombogenesis, further contributing to cardiorenal protection [31,32]. All these mechanisms that imply a complex interplay among cardiovascular, kidney, and metabolic systems could explain a self-perpetuating cycle of organ damage. Dysfunction in one of these systems may exacerbate pathology in the other, accelerating disease progression and increasing mortality risk [33].

SGLT-2is and GLP-1ras could play a central therapeutic role in patients with CKM syndrome by improving outcomes in each domain and addressing the pathophysiological links among them. Combination therapy with both an SGLT-2i and a GLP-1ra may be considered to provide the complementary outcomes benefits associated with these classes of medication, which are largely independent of glycemic management, making them foundational therapies for CKM syndrome [35]. While direct randomized controlled trials comparing combination therapy to monotherapy are lacking, the totality of evidence from subgroup analyses of cardiovascular outcome trials and large observational studies supports the use of both agents together to optimize cardiovascular and kidney outcomes in patients with CKM syndrome [36]. Furthermore, other therapies with renoprotective and cardioprotective effects, such as renin-angiotensin-aldosterone system inhibitors, statins, icosapent ethyl, and finerenone, could also contribute to managing patients with CKM syndrome according to their disease stage and comorbidities [21,37,38].

Lifestyle modification is the foundation of CKM syndrome management across all stages of the disease. Evidence supports dietary modification, increased physical activity, and behavioral interventions as first-line therapy, with intensive weight loss prioritized for those with obesity. These interventions are effective in both preventing and slowing progression of CKM syndrome [2].

Management of CKM syndrome must be guided by several core principles: holistic, multidisciplinary, and patient-centered care; integration of primary prevention and risk stratification; and explicit attention to social determinants of health. Early recognition and coordinated management—often led by primary care—are essential to prevent progression and reduce morbidity and mortality [2,39,40]. This management approach may be particularly relevant in older patients with HF with preserved LVEF, longer diabetes duration, poorer glycemic control, and more advanced renal impairment, according to findings from our exploratory subgroup analyses.

Despite recent advances, important gaps remain in the management of patients with CKM syndrome. Future research should focus on prospective, adequately powered studies assessing long-term cardiovascular and renal outcomes, as well as the validation and refinement of risk stratification tools in diverse CKM populations. In addition, further work is needed to evaluate differential treatment effects across clinically relevant subgroups and to develop and test implementation strategies aimed at reducing disparities in access to and delivery of care.

The results of this study are noteworthy, as it is the first analysis to provide relevant evidence regarding the impact of semaglutide 1.00 mg once weekly on adverse outcomes in patients with CKM syndrome. Nevertheless, these findings should be interpreted in light of several limitations. First, although PSM was used, confounding variables were adjusted for, and baseline characteristics were largely comparable between groups, the observational design inherently limits the ability to fully rule out the influence of unmeasured confounding factors. The only baseline imbalance observed was a higher proportion of control patients with HbA1c levels below 7% prior to matching. This difference could be largely explained by the reimbursement criteria established by Spain’s National Health System, which restricts funding for semaglutide to patients with HbA1c levels above 7% despite treatment with at least one glucose-lowering agent for a minimum of three months and a body mass index of 30 kg/m^2^ or higher; patients who do not meet these criteria must pay for the treatment themselves. As patients in the control group could receive dipeptidyl peptidase-4 inhibitors, which also act on the incretin pathway, the benefits associated with semaglutide may have been underestimated. Additionally, the subgroup analyses were exploratory; therefore, their results should be interpreted with caution. Second, while the number of primary outcome events was sufficient, the relatively low incidence of certain secondary outcomes limits the strength of the conclusions that can be drawn regarding their association with semaglutide use. Additionally, the follow-up duration was pragmatically defined in accordance with real-world data availability rather than an event-driven design. Although this approach reflects routine clinical practice, the low number of events for outcomes such as cardiovascular and all-cause mortality may have limited the statistical power to detect significant differences. Longer follow-up may be required to better assess these endpoints. Third, as the HF therapy could be adjusted at the attending physicians’ discretion during follow-up, the observed outcomes cannot be attributed exclusively to starting semaglutide 1.00 mg once weekly. Moreover, the results may have also been influenced by lifestyle recommendations, including advice on diet and physical activity tailored to each patient’s functional status, which was provided throughout the study period. Treatment adherence was not formally assessed, which may have further influenced the findings. Finally, only once-weekly semaglutide was assessed in this analysis. This choice reflects its widespread use in the clinical setting and the intention to evaluate its effects independently, without potential confounding variables from other GLP-1ras. Consequently, these findings should not be generalized to other agents within the GLP-1ra class.

## 4. Materials and Methods

### 4.1. Study Design and Patients

This work is a retrospective, observational, real-world study on patients with HF, CKD, obesity, and T2DM treated with semaglutide 1.00 mg once weekly (Sema-CKM Group) and patients not treated with semaglutide or any other GLP-1ra (Control-CKM Group) who have been followed up on for 24 months in the HF unit between June 2019 and December 2025. The inclusion criteria were: (i) diagnosis of HF, defined as the presence of signs and symptoms of HF with evidence of structural and/or functional heart abnormalities and/or increase in natriuretic peptides; (ii) diagnosis of CKD, defined as an estimated glomerular filtration rate (eGFR) < 60 mL/min/1.73 m^2^ or a urine albumin-to-creatinine ratio ≥ 30 mg/g; (iii) diagnosis of obesity, defined as a body mass index (BMI) greater than or equal to 30; (iv) diagnosis of T2DM, ascertained through a T2DM diagnosis in the medical records. The exclusion criteria were patients on dialysis, transplanted patients, those with an eGFR < 15 mL/min/1.73 m^2^, or persistent macroalbuminuria at baseline. All medical records were reviewed by two different investigators to confirm the diagnoses. Baseline characteristics, covariates, and outcome measures were extracted from routinely collected data available in the electronic health records of our public healthcare system. Patients who did not meet the inclusion criteria or did not provide written informed consent for the consultation of medical records were excluded.

Patients in the Sema-CKM Group started a once-weekly 0.25 mg dose of semaglutide for four weeks, which was able to be increased to 0.50 mg for the following four weeks until they reached the maintenance dose of 0.50 mg or 1.00 mg, according to the healthcare professionals’ clinical judgment. During follow-up, all patients received general recommendations on a healthy diet and physical activity according to their functional class and following recommendations from the HF and CKD guidelines [18,19]. Treatments with lipid-lowering drugs, antihypertensive agents, and diuretics were modified, if necessary, as per the healthcare professionals’ judgment.

Follow-up was conducted every three to four months as part of routine clinical care in the HF unit. Data on anthropometric (body weight, BMI, and waist circumference), sociodemographic, clinical (T2DM duration and treatment, principal cause of HF, HF duration, left ventricular ejection fraction (LVEF), previous medical history, and medication), and laboratory variables (serum creatinine, eGFR measured using the Chronic Kidney Disease Epidemiology Collaboration (CKD-EPI) equation, Ref. [41] basal fasting blood glucose (BG), glycated hemoglobin (HbA1c), LDL cholesterol, HDL cholesterol, total cholesterol, triglycerides, uric acid, hematocrit, N-terminal pro-brain natriuretic peptide (NT-pro-BNP), and urinary albumin/creatinine ratio) were collected at each evaluation. All anthropometric measurements were obtained using standard clinical procedures routinely applied in our center. Body weight, height, and waist circumference were measured by trained healthcare personnel following established protocols, using calibrated equipment available in the HF unit. Laboratory parameters were determined in the hospital’s central laboratory using validated and standardized analytical methods, in accordance with routine clinical practice. All assays were performed with certified equipment subject to regular quality control procedures, ensuring accuracy and reliability of the measurements. The total symptom score on the Spanish version of the Kansas City Cardiomyopathy Questionnaire (KCCQ) [42] was used to estimate HF health status. Adverse drug reactions, the need to discontinue semaglutide due to adverse events, HF events, all-cause hospitalizations, and death (all-cause and cardiovascular) were also recorded.

The study was approved by the Institutional Research Ethics Committee of Málaga (Code: REDI-v3-25-14/01/2019). Written informed consent for the consultation of medical records was obtained from all participants. This study was conducted in accordance with the Declaration of Helsinki. Data confidentiality and patient anonymity were maintained at all times.

### 4.2. Outcome Measures

The two primary outcomes were HF events (defined as a composite of emergency department visits due to HF decompensation, HF hospitalizations, and unplanned outpatient visits) and major kidney disease events (a composite of the onset of kidney failure—dialysis, transplantation, or eGFR < 15 mL/min/1.73 m^2^, a reduction of at least 50% in the eGFR from baseline, persistent macroalbuminuria, or death from kidney-related or cardiovascular causes) at 24 months. Secondary outcomes included HF health status (defined as a ≥5 point difference in the Spanish version of the KCCQ total symptom score), cardiovascular death, all-cause death, and all-cause hospitalizations. Glycemic control (determined by a reduction in HbA1c) and changes in body weight were also evaluated.

### 4.3. Statistical Analyses

Patient characteristics were analyzed using descriptive statistics: means ± standard deviation for continuous variables and absolute values and percentages for categorical variables. The two-sample Student’s *t*-test or the Mann–Whitney–Wilcoxon rank-sum test for continuous variables and Pearson’s chi-square test for categorical variables were used to determine differences between groups. The Pearson correlation coefficient was calculated to estimate linear correlations.

Patients were grouped according to the use of semaglutide. Propensity score matching (PSM) with a caliper of 0.2 and a greedy matching algorithm was used to match each patient who started semaglutide with a patient in the control group in a 1:1 manner and aligning index dates to ensure comparability in calendar time. The probability of starting semaglutide was estimated using a logistic regression model that included variables that could have affected treatment assignment or outcomes as independent variables (sex, age, anthropometric characteristics, previous medical history, patient characteristics, and laboratory findings). PSM adequacy was assessed using the post-PSM standardized difference of patient characteristics. A significant imbalance in the group was defined as a standardized difference > 10% between baseline variables.

Mixed-effect logistic regressions were used and adjusted for confounding variables (sociodemographic and clinical characteristics) included in the models to evaluate the association between treatment and study outcomes. Univariate and multivariate logistic regression models adjusted with confounding variables to estimate the treatment effect using the totality of the data were also performed as a sensitivity analysis. The change in body weight was evaluated via analysis of covariance, with the change in the endpoint at 24 months as the dependent variable. The regression analysis values were expressed as odds ratios (OR) and 95% confidence intervals (95% CI). Deaths were treated as clinical outcomes, whereas loss to follow-up was defined as failure to complete follow-up for reasons other than death. Statistical significance was defined as *p* < 0.05. Statistical analyses were performed using SPSS Statistics for Windows, version 22.0 (IBM SPSS Statistics for Windows, IBM Corporation, Armonk, NY, USA).

## 5. Conclusions

Semaglutide 1.00 mg once weekly was associated with reductions in HF events and their individual components (emergency department visits for HF decompensation, HF hospitalizations, and unplanned outpatient visits) as well as major kidney disease events in patients with CKM syndrome. There was also an improvement in HF health status and a reduction in all-cause hospitalizations. The tolerability and safety profile of semaglutide were good. Further research on GLP-1ras in CKM syndrome is needed and results from long-term randomized clinical trials are required to provide more evidence on their efficacy and safety and to refine risk stratification.

## Figures and Tables

**Table 1:** Sociodemographic, clinical, and therapeutic characteristics at baseline: pre- and post-propensity score matching analysis.

	Pre-Propensity Score Matching Analysis				Post-Propensity Score Matching Analysis			
Variables	Sema-CKM Group (n = 388)	Control-CKM Group (n = 366)	SD	*p*-Value	Sema-CKM Group (n = 302)	Control-CKM Group (n = 302)	SD	*p*-Value
Sociodemographic characteristics								
Age (years)	74.8 (10.1)	76.2 (11.4)	0.031	0.163	75.5 (11.0)	76.0 (11.1)	0.024	0.224
Female	198 (51.0%)	201 (54.9%)	0.065	0.145	158 (52.3%)	161 (53.3%)	0.038	0.206
Anthropometric characteristics								
Body weight (kg)	97.0 (16.0)	93.7 (13.5)	0.080	0.105	95.9 (15.1)	94.5 (14.9)	0.048	0.192
Body Mass Index (kg/m^2^)	35.5 (4.9)	32.0 (1.7)	0.088	0.089	34.5 (4.4)	32.9 (2.1)	0.072	0.139
Body Mass Index ≥ 30 kg/m^2^	388 (100.0%)	366 (100.0%)	0.009	0.299	302 (100.0%)	302 (100.0%)	0.005	0.301
Waist circumference (cm)	132.9 (15.8)	128.0 (14.2)	0.029	0.199	132.0 (15.4)	129.9 (14.4)	0.021	0.204
SBP (mmHg)	134.0 (14.0)	130.0 (11.6)	0.084	0.101	133.5 (13.0)	132.0 (12.0)	0.061	0.184
DBP (mmHg)	72.5 (9.9)	70.0 (8.0)	0.039	0.212	72.0 (9.5)	70.5 (8.5)	0.018	0.255
Heart rate (bpm)	72.0 (10.0)	70.0 (9.0)	0.015	0.275	72.0 (10.0)	71.0 (9.0)	0.014	0.283
Diabetes characteristics								
Diabetes duration (years)	15.0 (7.5)	13.0 (7.0)	0.069	0.154	14.5 (7.3)	13.5 (7.0)	0.040	0.192
Patients with HbA1c < 7%	14 (3.6%)	73 (19.9%)	0.133	0.015	14 (4.6%)	29 (9.6%)	0.079	0.092
Diabetes therapy			0.065	0.143			0.021	0.207
Metformin	225 (58.0%)	163 (59.5%)			177 (58.6%)	178 (58.9%)		
Sulfonylurea	3 (0.8%)	3 (0.8%)			2 (0.6%)	2 (0.6%)		
DPP-4 inhibitor	156 (40.2%)	165 (45.1%)			127 (42.1%)	131 (43.4%)		
GLP-1 receptor agonist (not semaglutide)	100 (25.8%)	0			77 (25.5%)	0		
SGLT-2 inhibitor	214 (55.2%)	191 (52.2%)			166 (55.0%)	162 (53.6%)		
Basal insulin	132 (34.0%)	135 (36.9%)			105 (34.8%)	107 (35.4%)		
Basal insulin dose (Units/day)	19.4 ± 12.5	21.0 ± 14.1			20.0 ± 12.6	21.0 ± 14.0		
Insulin combinations	35 (9.0%)	37 (10.1%)			28 (9.3%)	29 (9.6%)		
Statins	360 (92.8%)	344 (94.0%)	0.030	0.228	281 (93.0%)	283 (93.7%)	0.021	0.252
Heart failure characteristics								
Heart failure duration (years)	7.9 (3.7)	8.4 (3.9)	0.051	0.159	8.0 (3.8)	8.2 (3.8)	0.030	0.199
Principal cause of heart failure			0.041	0.188			0.029	0.201
Ischemic	98 (25.3%)	80 (21.9%)			70 (23.2%)	68 (22.5%)		
Non-ischemic	290 (74.7%)	286 (78.1%)			232 (76.8%)	234 (77.5%)		
KCCQ total symptom score	51.5 (21.0)	55.5 (23.5)	0.040	0.191	53.5 (22.5)	55.0 (23.0)	0.017	0.268
NYHA functional class			0.068	0.138			0.030	0.198
II	213 (54.9%)	220 (60.1%)			167 (55.3%)	170 (56.3%)		
III	175 (45.1%)	146 (39.9%)			135 (44.7%)	132 (43.7%)		
Left ventricular ejection fraction (%)	58.0 (10.5)	59.9 (13.0)	0.010	0.299	58.0 (10.8)	59.5 (13.0)	0.006	0.301
Left ventricular ejection fraction ≤ 40%	97 (25.0%)	81 (22.1%)	0.023	0.259	73 (24.2%)	70 (23.2%)	0.010	0.298
Heart failure medication			0.038	0.169			0.031	0.199
Diuretic	350 (90.2%)	340 (92.9%)			274 (90.7%)	275 (91.1%)		
ACE inhibitor	63 (16.2%)	66 (18.0%)			51 (16.9%)	53 (17.5%)		
ARB	156 (40.2%)	140 (38.3%)			120 (39.7%)	119 (39.4%)		
Sacubitril-valsartan	85 (21.9%)	73 (19.9%)			63 (20.9%)	62 (20.5%)		
Beta-blocker	252 (64.9%)	219 (59.8%)			188 (62.3%)	184 (60.9%)		
Ivabradine	47 (12.1%)	54 (14.8%)			39 (12.9%)	41 (13.6%)		
Mineralocorticoid receptor antagonist	97 (25.0%)	85 (23.2%)			75 (24.8%)	73 (24.2%)		
Digitalis	11 (2.8%)	9 (2.5%)			8 (2.6%)	8 (2.6%)		
Anticoagulant	199 (51.3%)	179 (48.9%)			152 (50.3%)	150 (49.7%)		
Previous medical history								
History of smoking	209 (53.9%)	191 (52.2%)	0.050	0.148	160 (53.0%)	159 (52.6%)	0.029	0.202
History of alcohol use disorder	39 (10.1%)	33 (9.0%)	0.034	0.195	28 (9.3%)	29 (9.6%)	0.025	0.209
Hypertension	381 (98.2%)	348 (95.1%)	0.025	0.224	295 (97.7%)	290 (96.0%)	0.010	0.299
Dyslipidemia	357 (92.0%)	248 (95.1%)	0.045	0.184	281 (93.0%)	285 (94.4%)	0.024	0.208
Cerebrovascular disease	58 (14.9%)	37 (10.1%)	0.049	0.149	39 (12.9%)	35 (11.6%)	0.035	0.190
Chronic obstructive pulmonary disease	174 (44.8%)	147 (40.2%)	0.055	0.145	129 (42.7%)	125 (41.4%)	0.019	0.273
Atrial fibrillation	202 (52.1%)	182 (49.7%)	0.060	0.135	157 (52.0%)	154 (51.0%)	0.029	0.224
Laboratory variables								
Glucose (mg/dL)	151.0 (42.5)	141.5 (36.5)	0.095	0.064	150.0 (42.0)	144.0 (39.0)	0.090	0.099
HbA1c (%)	8.1 (1.5)	7.2 (1.1)	0.107	0.039	7.8 (1.3)	7.3 (1.1)	0.064	0.085
Creatinine (mg/dL)	1.7 (0.5)	1.8 (0.5)	0.025	0.197	1.7 (0.5)	1.7 (0.5)	0.016	0.228
EGFR (mL/min/1.73 m^2^)	49.5 (23.0)	51.5 (24.5)	0.026	0.195	50.0 (23.2)	51.0 (24.2)	0.012	0.249
Uric acid (mg/dL)	7.8 (5.0)	6.5 (4.5)	0.038	0.191	7.5 (4.9)	6.9 (4.7)	0.018	0.222
Hematocrit (%)	38.0 (4.5)	36.2 (4.0)	0.024	0.198	37.5 (4.2)	36.5 (4.1)	0.020	0.212
NT-proBNP (pg/mL)	1245.0 (582.0)	1050.5 (530.0)	0.048	0.158	1150.5 (552.0)	1098.5 (541.0)	0.035	0.195
LDL cholesterol (mg/dL)	72.5 (25.5)	75.5 (27.5)	0.029	0.185	73.5 (25.9)	75.0 (27.4)	0.017	0.218
HDL cholesterol (mg/dL)	40.5 (9.5)	43.5 (9.9)	0.036	0.175	41.5 (9.7)	42.5 (9.8)	0.033	0.221
Total cholesterol (mg/dL)	155.0 (36.5)	149.5 (33.5)	0.043	0.165	152.5 (35.5)	151.0 (35.0)	0.018	0.225
Triglycerides (mg/dL)	185.0 (50.5)	200.2 (55.5)	0.045	0.161	189.0 (53.5)	195.5 (55.0)	0.030	0.196
Urinary albumin/creatinine ratio (mg/g)	218.5 (60.5)	257.0 (73.5)	0.077	0.114	233.5 (65.5)	249.5 (69.8)	0.035	0.188

Continuous data are shown as means (standard deviations) and qualitative data as absolute values and percentages. A significant imbalance in the group was defined as a standardized difference > 10% between baseline variables. Statistical significance was defined as *p* < 0.05. ACE: angiotensin-converting enzyme; ARB: angiotensin receptor blocker; DBP: diastolic blood pressure; DPP4: dipeptidyl peptidase-4; EGFR: estimated glomerular filtration rate; GLP-1: glucagon-like peptide-1; HbA1c: glycated hemoglobin; HF: heart failure; KCCQ: Kansas City Cardiomyopathy Questionnaire; NT-proBNP: N-terminal pro-brain natriuretic peptide; NYHA: New York Heart Association; SBP: systolic blood pressure; SD: standardized difference; SGLT-2: sodium–glucose cotransporter 2.

## Data Availability

The original contributions presented in this study are included in the article. Further inquiries can be directed to the corresponding author.

## References

[1] Kittelson K.S., Junior A.G., Fillmore N., da Silva Gomes R. (2024). Cardiovascular-kidney metabolic syndrome—An integrative review. Prog. Cardiovasc. Dis..

[2] Ferdinand K.C. (2024). An overview of cardiovascular-kidney-metabolic syndrome. Am. J. Manag. Care.

[3] Gómez-Huelgas R., Sanz-Cánovas J., Cobos-Palacios L., López-Sampalo A., Pérez-Belmonte L.M. (2022). Glucagon-like peptide-1 receptor agonists and sodium-glucose cotransporter 2 inhibitors for cardiovascular and renal protection: A treatment approach far beyond their glucose-lowering effect. Eur. J. Intern. Med..

[4] Patel S.M., Kang Y.M., Im K., Neuen B.L., Anker S.D., Bhatt D.L., Butler J., Cherney D.Z., Claggett B.L., Fletcher R.A. (2024). Sodium-Glucose Cotransporter-2 Inhibitors and Major Adverse Cardiovascular Outcomes: A SMART-C Collaborative Meta-Analysis. Circulation.

[5] Kristensen S.L., Rørth R., Jhund P.S., Docherty K.F., Sattar N., Preiss D., Køber L., Petrie M.C., McMurray J.J.V. (2019). Cardiovascular, mortality, and kidney outcomes with GLP-1 receptor agonists in patients with type 2 diabetes: A systematic review and meta-analysis of cardiovascular outcome trials. Lancet Diabetes Endocrinol..

[6] Kosiborod M.N., Petrie M.C., Borlaug B.A., Butler J., Davies M.J., Hovingh G.K., Kitzman D.W., Møller D.V., Treppendahl M.B., Verma S. (2024). Semaglutide in Patients with Obesity-Related Heart Failure and Type 2 Diabetes. N. Engl. J. Med..

[7] Kosiborod M.N., Abildstrøm S.Z., Borlaug B.A., Butler J., Rasmussen S., Davies M., Hovingh G.K., Kitzman D.W., Lindegaard M.L., Møller D.V. (2023). Semaglutide in Patients with Heart Failure with Preserved Ejection Fraction and Obesity. N. Engl. J. Med..

[8] Qin W., Yang J., Ni Y., Deng C., Ruan Q., Ruan J., Zhou P., Duan K. (2024). Efficacy and safety of once-weekly tirzepatide for weight management compared to placebo: An updated systematic review and meta-analysis including the latest SURMOUNT-2 trial. Endocrine.

[9] Packer M., Zile M.R., Kramer C.M., Baum S.J., Litwin S.E., Menon V., Ge J., Weerakkody G.J., Ou Y., Bunck M.C. (2025). Tirzepatide for Heart Failure with Preserved Ejection Fraction and Obesity. N. Engl. J. Med..

[10] McMurray J.J.V., Solomon S.D., Inzucchi S.E., Køber L., Kosiborod M.N., Martinez F.A., Ponikowski P., Sabatine M.S., Anand I.S., Bělohlávek J. (2019). Dapagliflozin in patients with heart failure and reduced ejection fraction. N. Engl. J. Med..

[11] Packer M., Anker S.D., Butler J., Filippatos G., Pocock S.J., Carson P., Januzzi J., Verma S., Tsutsui H., Brueckmann M. (2020). Cardiovascular and renal outcomes with empagliflozin in heart failure. N. Engl. J. Med..

[12] Anker S.D., Butler J., Filippatos G., Ferreira J.P., Bocchi E., Böhm M., Brunner–La Rocca H.-P., Choi D.-J., Chopra V., Chuquiure-Valenzuela E. (2021). Empagliflozin in Heart Failure with a Preserved Ejection Fraction. N. Engl. J. Med..

[13] Solomon S.D., McMurray J.J., Claggett B., de Boer R.A., DeMets D., Hernandez A.F., Inzucchi S.E., Kosiborod M.N., Lam C.S., Martinez F. (2022). Dapagliflozin in Heart Failure with Mildly Reduced or Preserved Ejection Fraction. N. Engl. J. Med..

[14] Perkovic V., Jardine M.J., Neal B., Bompoint S., Heerspink H.J.L., Charytan D.M., Edwards R., Agarwal R., Bakris G., Bull S. (2019). Canagliflozin and Renal Outcomes in Type 2 Diabetes and Nephropathy. N. Engl. J. Med..

[15] Heerspink H.J.L., Stefánsson B.V., Correa-Rotter R., Chertow G.M., Greene T., Hou F.-F., Mann J.F.E., McMurray J.J.V., Lindberg M., Rossing P. (2020). Dapagliflozin in Patients with Chronic Kidney Disease. N. Engl. J. Med..

[16] Bhatt D.L., Szarek M., Pitt B., Cannon C.P., Leiter L.A., McGuire D.K., Lewis J.B., Riddle M.C., Inzucchi S.E., Kosiborod M.N. (2021). Sotagliflozin in Patients with Diabetes and Chronic Kidney Disease. N. Engl. J. Med..

[17] Herrington W.G., Staplin N., Wanner C., Green J.B., Hauske S.J., Emberson J.R., Preiss D., Judge P., Mayne K.J., The EMPA-KIDNEY Collaborative Group (2023). Empagliflozin in Patients with Chronic Kidney Disease. N. Engl. J. Med..

[18] McDonagh T.A., Metra M., Adamo M., Gardner R.S., Baumbach A., Böhm M., Burri H., Butler J., Čelutkienė J., Chioncel O. (2022). 2021 ESC Guidelines for the diagnosis and treatment of acute and chronic heart failure: Developed by the Task Force for the diagnosis and treatment of acute and chronic heart failure of the European Society of Cardiology (ESC). With the special contribution of the Heart Failure Association (HFA) of the ESC. Eur. J. Heart Fail..

[19] (2024). Kidney Disease: Improving Global Outcomes (KDIGO) CKD Work Group. KDIGO 2024 Clinical Practice Guideline for the Evaluation and Management of Chronic Kidney Disease. Kidney Int..

[20] Akiyama H., Nishimura A., Morita N., Yajima T. (2023). Evolution of sodium-glucose co-transporter 2 inhibitors from a glucose-lowering drug to a pivotal therapeutic agent for cardio-renal-metabolic syndrome. Front. Endocrinol..

[21] Pohlman N., Patel P.N., Essien U.R., Tang J.J., Joseph J.J. (2025). Novel Cardiometabolic Medications in the Cardiovascular-Kidney-Metabolic Syndrome Era. J. Clin. Endocrinol. Metab..

[22] Perkovic V., Tuttle K.R., Rossing P., Mahaffey K.W., Mann J.F., Bakris G., Baeres F.M., Idorn T., Bosch-Traberg H., Lausvig N.L. (2024). Effects of Semaglutide on Chronic Kidney Disease in Patients with Type 2 Diabetes. N. Engl. J. Med..

[23] Lincoff A.M., Brown-Frandsen K., Colhoun H.M., Deanfield J., Emerson S.S., Esbjerg S., Hardt-Lindberg S., Hovingh G.K., Kahn S.E., Kushner R.F. (2023). Semaglutide and Cardiovascular Outcomes in Obesity without Diabetes. N. Engl. J. Med..

[24] Navaneethan S.D., Bansal N., Cavanaugh K.L., Chang A., Crowley S., Delgado C., Estrella M.M., Ghossein C., Ikizler T.A., Koncicki H. (2025). KDOQI US Commentary on the KDIGO 2024 Clinical Practice Guideline for the Evaluation and Management of CKD. Am. J. Kidney Dis..

[25] Pratley R.E., Tuttle K.R., Rossing P., Rasmussen S., Perkovic V., Nielsen O.W., Mann J.F., MacIsaac R.J., Kosiborod M.N., Kamenov Z. (2024). Effects of Semaglutide on Heart Failure Outcomes in Diabetes and Chronic Kidney Disease in the FLOW Trial. J. Am. Coll. Cardiol..

[26] Gerstein H.C., Sattar N., Rosenstock J., Ramasundarahettige C., Pratley R., Lopes R.D., Lam C.S., Khurmi N.S., Heenan L., Del Prato S. (2021). Cardiovascular and Renal Outcomes with Efpeglenatide in Type 2 Diabetes. N. Engl. J. Med..

[27] Pérez-Belmonte L.M., Sanz-Cánovas J., de Lucas M.D.G., Ricci M., Avilés-Bueno B., Cobos-Palacios L., Pérez-Velasco M.A., López-Sampalo A., Bernal-López M.R., Jansen-Chaparro S. (2022). Efficacy and Safety of Semaglutide for the Management of Obese Patients with Type 2 Diabetes and Chronic Heart Failure in Real-World Clinical Practice. Front. Endocrinol..

[28] Pérez-Velasco M.A., Trenas A., Bernal-López M.R., García de Lucas M.D., Gómez-Huelgas R., Pérez-Belmonte L.M. (2024). Once weekly semaglutide and cardiovascular outcomes in patients with type 2 diabetes and heart failure with reduced left ventricular ejection fraction. Rev. Esp. Cardiol. (Engl. Ed.).

[29] Pérez-Velasco M.A., Bernal-López M.-R., Trenas A., Ricci M., López-Carmona M.-D., de Lucas M.-D.G., Gómez-Huelgas R., Pérez-Belmonte L.M. (2025). Efficacy of once-weekly semaglutide in patients with heart failure with preserved ejection fraction, obesity and type 2 diabetes. Med. Clin..

[30] Trenas A., Pérez-Velasco M.A., Bernal-López M.-R., López-Carmona M.-D., Gómez-Doblas J.J., Martínez-Esteban M.-D., Bouarich O., García-Casares N., Fernández-García D., de Lucas M.-D.G. (2025). Efficacy and safety of once-daily oral semaglutide in patients with heart failure with preserved ejection fraction, type 2 diabetes and obesity: A real-world study. Eur. J. Intern. Med..

[31] Ussher J.R., Drucker D.J. (2023). Glucagon-like peptide 1 receptor agonists: Cardiovascular benefits and mechanisms of action. Nat. Rev. Cardiol..

[32] Tommerdahl K.L., Nadeau K.J., Bjornstad P. (2021). Mechanisms of Cardiorenal Protection of Glucagon-Like Peptide-1 Receptor Agonists. Adv. Chronic Kidney Dis..

[33] Nauck M.A., Tuttle K.R., Tschöp M.H., Blüher M. (2026). Glucagon-like receptor agonists and next-generation incretin-based medications: Metabolic, cardiovascular, and renal benefits. Lancet.

[34] Thomas M.C., Coughlan M.T., Cooper M.E. (2023). The postprandial actions of GLP-1 receptor agonists: The missing link for cardiovascular and kidney protection in type 2 diabetes. Cell Metab..

[35] American Diabetes Association Professional Practice Committee for Diabetes (2026). Chronic Kidney Disease and Risk Management: Standards of Care in Diabetes-2026. Diabetes Care.

[36] Apperloo E.M., Neuen B.L., Fletcher R.A., Jongs N., Anker S.D., Bhatt D.L., Butler J., Cherney D.Z.I., Herrington W.G., Inzucchi S.E. (2024). Efficacy and safety of SGLT2 inhibitors with and without glucagon-like peptide 1 receptor agonists: A SMART-C collaborative meta-analysis of randomised controlled trials. Lancet Diabetes Endocrinol..

[37] Ndumele C.E., Neeland I.J., Tuttle K.R., Chow S.L., Mathew R.O., Khan S.S., Coresh J., Baker-Smith C.M., Carnethon M.R., Després J.-P. (2023). A Synopsis of the Evidence for the Science and Clinical Management of Cardiovascular-Kidney-Metabolic (CKM) Syndrome: A Scientific Statement From the American Heart Association. Circulation.

[38] Alicic R.Z., Neumiller J.J., Tuttle K.R. (2025). Combination therapy: An upcoming paradigm to improve kidney and cardiovascular outcomes in chronic kidney disease. Nephrol. Dial. Transplant..

[39] Orozco-Beltrán D., Quiroga B., Esteban-Fernández A., Almorós A.L., Bellido V., de Inestrosa T.B.P., de Haro R., Taboada X., Romero-Vigara J.C. (2025). Evaluation, Management and Therapeutic Approach of Cardiovascular-Kidney-Metabolic Syndrome: A Multidisciplinary Delphi Expert Consensus. J. Clin. Med..

[40] Gunnarsson S., Vito O., Unwin R.J. (2026). Cardiovascular-kidney-metabolic syndrome: Prevalence, risks, disease trajectories, and early-stage management. Am. J. Physiol. Cell Physiol..

[41] Valente M.A., Hillege H.L., Navis G., Voors A.A., Dunselman P.H., van Veldhuisen D.J., Damman K. (2014). The Chronic Kidney Disease Epidemiology Collaboration equation outperforms the Modification of Diet in Renal Disease equation for estimating glomerular filtration rate in chronic systolic heart failure. Eur. J. Heart Fail..

[42] Comín-Colet J., Garin O., Lupón J., Manito N., Crespo-Leiro M.G., Gómez-Bueno M., Ferrer M., Artigas R., Zapata A., Elosua R. (2011). Validation of the Spanish version of the Kansas city cardiomyopathy questionnaire. Rev. Esp. Cardiol..

